# Adapting a Social Prescribing Decision Support Tool for Community and Health Organizations

**DOI:** 10.1093/geroni/igaf122.1574

**Published:** 2025-12-31

**Authors:** Ashwin Kotwal, Carla Perissinotto, Katrina Hough, Nandini Singh, Shannon Fuller

**Affiliations:** University of California, San Francisco, San Francisco, California, United States; University of California, San Francisco, San Francisco, California, United States; University of California, San Francisco, San Francisco, California, United States; University of California, San Francisco, San Francisco, California, United States; Johns Hopkins University, Baltimore, Maryland, United States

## Abstract

Social connection is a critical determinant of health, yet loneliness and social isolation remain pervasive challenges among older adults. Social prescribing—an evidence-based approach to matching individuals with social interventions tailored to their needs—holds promise in bridging the gap between healthcare and community-based services. However, there is a lack of structured, cross-sector tools to support this process. This presentation describes the initial steps of a community-engaged initiative to adapt and implement a Social Prescribing Decision Support Tool for use across diverse settings, including senior centers, health systems, and community-based organizations. Guided by a social-ecological conceptual framework, we plan to use qualitative methods and implementation science to adapt an existing Social Prescribing Decision Support Tool through an iterative process with feedback from older adults, community program staff, healthcare providers, and national experts. The resulting tool will account for intake and referral workflows, as well as the capacity to implement social prescribing in each setting. It will facilitate structured assessment and individualized matching of older adults to social interventions that address key dimensions of social connection (structure, function, and quality). We will present preliminary findings from the adaptation process, discuss lessons learned from community partnerships, and outline plans for a pilot implementation in two community-based organizations. This work aims to advance community-engaged scholarship by co-designing scalable, pragmatic solutions that enhance social prescribing efforts, promote interdisciplinary collaboration, and improve social well-being outcomes for older adults.

